# Deficits in Long-Term Recognition Memory Reveal Dissociated Subtypes in Congenital Prosopagnosia

**DOI:** 10.1371/journal.pone.0015702

**Published:** 2011-01-25

**Authors:** Rainer Stollhoff, Jürgen Jost, Tobias Elze, Ingo Kennerknecht

**Affiliations:** 1 Max Planck Institute for Mathematics in the Sciences, Leipzig, Germany; 2 Santa Fe Institute for the Sciences of Complexity, Santa Fe, New Mexico, United States of America; 3 Institute of Human Genetics, Westfälische Wilhelms-Universität, Münster, Germany; National Institute of Mental Health, United States

## Abstract

The study investigates long-term recognition memory in congenital prosopagnosia (CP), a lifelong impairment in face identification that is present from birth. Previous investigations of processing deficits in CP have mostly relied on short-term recognition tests to estimate the scope and severity of individual deficits. We firstly report on a controlled test of long-term (one year) recognition memory for faces and objects conducted with a large group of participants with CP. Long-term recognition memory is significantly impaired in eight CP participants (CPs). In all but one case, this deficit was selective to faces and didn't extend to intra-class recognition of object stimuli. In a test of famous face recognition, long-term recognition deficits were less pronounced, even after accounting for differences in media consumption between controls and CPs. Secondly, we combined test results on long-term and short-term recognition of faces and objects, and found a large heterogeneity in severity and scope of individual deficits. Analysis of the observed heterogeneity revealed a dissociation of CP into subtypes with a homogeneous phenotypical profile. Thirdly, we found that among CPs self-assessment of real-life difficulties, based on a standardized questionnaire, and experimentally assessed face recognition deficits are strongly correlated. Our results demonstrate that controlled tests of long-term recognition memory are needed to fully assess face recognition deficits in CP. Based on controlled and comprehensive experimental testing, CP can be dissociated into subtypes with a homogeneous phenotypical profile. The CP subtypes identified align with those found in prosopagnosia caused by cortical lesions; they can be interpreted with respect to a hierarchical neural system for face perception.

## Introduction

Prosopagnosia refers to a selective deficit in the processing of facial identity [1]. Initial reports of prosopagnosia only covered cases where the deficit was acquired due to cortical lesions [1]–[9], see [10] for a review of 74 cases. Acquired prosopagnosia (AP) is a heterogeneous disorder, where the nature, extent, and selectivity of the deficit depend on the exact location of the lesion. Variations include a lack of overt recognition of familiar faces with intact covert recognition [8], [11]–[14], a deficit in configural encoding of faces (and objects) [15]–[17], an impaired imagery of faces [18], and difficulties in processing facial expressions [19]. But although in most cases the deficit is not restricted to facial identification, there are cases of AP with intact object recognition abilities [20].

During the last decades more and more cases of prosopagnosia have been reported where the impairment was not acquired due to an accident, but presumably present from birth [8], [21]–[29]. Congenital prosopagnosia (CP) is among the most common anomalies in humans with a prevalence of 2.5% [28], and is almost always hereditary [29]–[32], see Discussion.

The face recognition deficit in CP can be as profound as in the acquired form and equally selective such that only facial identification is impaired while all other aspects of face and object recognition remain intact [33], [34]. Similar to AP, cases often display heterogeneous symptoms [35] which might include additional impairments, e.g. in the processing of biological motion [36] or impaired visual mental imagery [37]. The observed heterogeneity in test results [35] has so far prevented a stringent categorization of CP according to more specific deficits in the processing of facial identity.

Previous characterizations of CP have been focused on

- dissociations between face and object recognition [33], [34],
- dissociations between face detection and face recognition [38],
- the processing of facial identity and facial expressions [19],
- global and local processing [39],
- holistic, configural, and featural processing [40], [41],
- processing of inverted and upright faces [27],
- differences in gaze behavior and eye-movements [42]

either testing single aspects in isolation, or by conducting a battery of tests with the same participants [27], [35], [39], [43]. Of the facial identification tests applied previously, most assessed short-term recognition memory in experimental settings. The exception are tests of familiar and of famous face recognition. However, these tests of long-term recognition suffer from either a limited comparability across different studies or a limited validity, as a bad performance in recognizing famous faces might result from a decreased social interest. Furthermore, investigations of CP conducted so far have mostly recruited only a small number of CP participants (n<10). In general, the developmental aspect of CP complicates a generalization from a small number of cases. Most CPs have evolved individual compensatory strategies to deal with their deficit. These compensatory processing strategies may or may not enable them to perform normally in behavioral experiments. The strategies adopted can vary greatly between individual prosopagnosics, which at least complicates a characterization of CP based on a small number of participants and/or behavioral tests.

In this study, we report on a detailed assessment of long- and short-term recognition memory for faces and individual objects in a larger number of CP participants (n = 15). Firstly, we conducted a test of long-term recognition memory for faces as well as object stimuli with a retention interval of one year. The results of this controlled tests were then compared to a test of famous face recognition to assess the validity of the latter. Secondly, we complemented the tests of long-term recognition memory with each participants results in short-term recognition tests conducted previously [44]. To enable a comparison of test results across experiments and to account for differences in possible confounding factors (e.g. age), we calculated abnormality scores measuring the deviation of each participant's performance from the statistical average.

### Long-term recognition memory in CP

In order to investigate long-term recognition memory for faces and objects in CP, we conducted an experiment which tested recognition performance after a retention interval of one year. To assess face specificity in potential deficits the experiment was conducted with face as well as object stimuli. As faces are normally distinguished on an individual level [45], [46], we constructed a set of individual shoes (Nike sneakers) as object stimuli. Using standardized, previously unfamiliar stimuli guaranteed that, in contrast to a famous face test, all participants had the same degree of familiarization.

In addition, we conducted a test of famous face recognition, the Bielefeld Famous Face Test (BFFT), originally developed to test for amnesia [47], [48]. On the one hand, famous faces tests directly draw on existing memories, thus limiting the applicability of ad-hoc compensatory strategies. On the other hand, differences in prior exposure to the faces introduce variability that is difficult to assess and control.

### Categorization of processing deficits

The second aim of this study is to investigate a symptomatic categorization of congenital prosopagnosia along the lines of an apperceptive, associative, or amnestic subtype [5], [49]. In the apperceptive subtype, the face recognition deficit is primarily due to a dysfunctional perceptual encoding of face images, in the associative subtype due to difficulties in associating encoded percepts with individual facial identities, and in the amnestic subtype the deficit is restricted to establishing and maintaining the long-term stability of an association between a facial identity and a semantic identity.

Here, the categorization will be based on performance of a large group of participants in a battery of behavioral tests, including measurements of reaction time under unlimited viewing, presentation time needed for accurate recognition, and performance if test images are rotated in depth [44]. All of the tests measured performance in either face or object identification; stimulus transformations (e.g. viewpoint, illumination) were restricted to natural stimulus transformations, i.e. no scrambling or inverting.

Individual tests were a priori grouped into tests of either perceptual, associative, or mnestic aspects, based on theoretical models of facial information processing [44], [50]–[53]. In contrast to an unsupervised a posteriori grouping of behavioral performance, e.g. based on principal component analysis [43], this intentional grouping provides a direct assessment of specific subcomponents and allows for intrinsic correlations. In cases of AP, correlations in the acquired deficits are primarily due to the extent of the lesion [49]. Although lesions are absent in CP correlations are also to be expected due to the hierarchical nature of face processing [52]. For example, an apperceptive deficit will always lead to compensatory, presumably suboptimal, associations. This hierarchical nesting of deficits complicates the application of unsupervised methods which aim at identifying uncorrelated deficit patterns.

## Results

### Long-term recognition memory

On average recognition of target images with a retention interval of one year was worse in CPs than in controls for faces but not for shoes. The difference in face recognition was mostly due to an increase in the miss rate, i.e. CPs failed to recognize the four target faces more often than controls.

**Figure 1:**
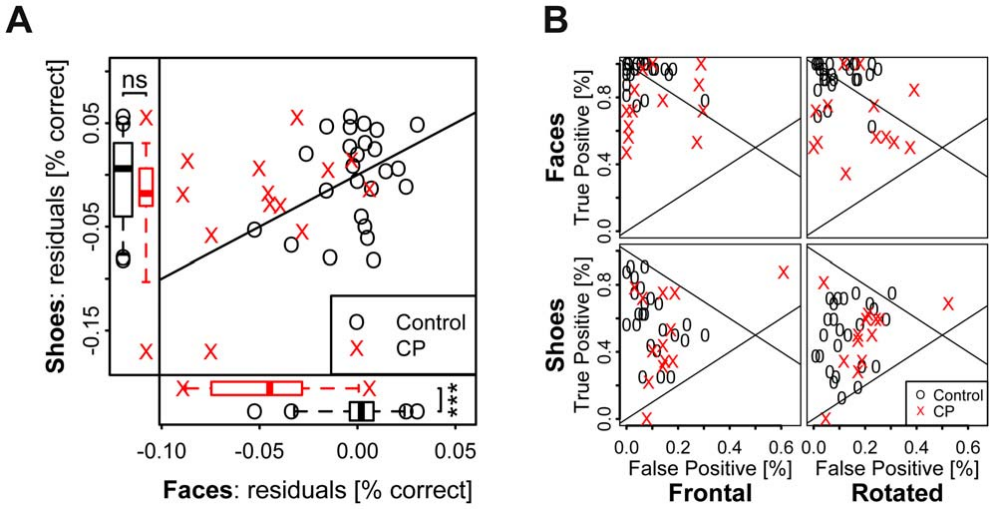
Face recognition memory for faces and shoes. (A) Compared to controls (O), CPs (X) showed worse recognition performance after one year for target faces but not for shoes. (B) The difference in face recognition is largely due to a decreased false positive rate among CPs who failed to detect the target faces more often than controls.

Testing for group differences in face recognition performance, we found that CPs, with a median error rate of 14.7%, perform significantly worse than controls, with a median of 6.3% (Wilcoxon rank sum test, W = 267, n_0_ = 25, n_CP_ = 13, p<0.001 one-sided). Separating the errors made during face recognition into false positives (false alarms) and false negatives (misses) revealed a disproportionally higher rate of misses among CPs (see figure 1 B). We therefore calculated d′ as a bias free measure of recognition performance. As a group, CPs showed significantly lower d′ values than controls (medians of 1.94 and 3.31 for CPs and controls; W = 186, n_0_ = 25, n_CP_ = 13, p<0.001 one-sided).

When testing for differences in the recognition of shoes, we observed higher error rates among CPs, median error rate of 24.1%, compared to controls, median of 18.4% (W = 223, n_0_ = 25, n_CP_ = 13, p = 0.032 one-sided). However, observed group differences in d′ are only borderline significant (medians of 0.70 and 1.46 for CPs and controls; W = 195, n_0_ = 25, n_CP_ = 13, p = 0.075 one-sided).

Due to differences in participation (see below), the mean age of CPs was 5.3 years older than for controls. To account for age-related differences in recognition performance, we fitted generalized linear mixed models (GLMMs) including age, trial type (target present or not present) and rotation (frontal or rotated) as fixed effects and participant identity as a random effect. Based on the models we calculated for each participant a performance residual as the difference between observed performance and the performance that would be expected of a control with identical age. Among CP participants residuals are larger than among controls for faces (Wilcoxon rank sum test, W = 286, n_0_ = 25, n_CP_ = 13, p<0.001 one-sided) but not for shoes (W = 204, n_0_ = 25, n_CP_ = 13, p = 0.11 one-sided). Thus, as a group, CPs performed worse than expected, given their age, only in the recognition of faces but not of shoes. Comparing each participant's residuals in face and shoe recognition we found that the deficit is selective for a majority CPs (see figure 1 A, residual values above the diagonal).

Tests for group differences, based on comparing the above described GLMMs, revealed a significant main effect in face recognition, i.e. a difference in the average performance, with CPs performing worse (LR-test of main effect against nullmodel, D = 11.94, df = 1, p<0.001; β_CP_ = −1.04, HPDI_95%_ = [−1.69, −0.62]). In the face recognition experiments, we also found significant group differences in the influence of trial type and image rotation on performance (LR-test of a full model, including interactions of group with trial type and rotation, against a main effect model: D = 7.97, df = 2, p = 0.019). Analysis of the full model revealed a significant difference in the influence of trial type: CPs made more mistakes in recognizing rotated target faces than controls (difference to controls: β_CP*trial_ = −1.63, HPDI_95%_ = [−2.28,−0.41]). In contrast, the influence of image rotation didn't differ between the two groups (β_CP*rotation_ = 0.31, HPDI_95%_ = [−0.88, 1.00]).

In the recognition of shoes, there was a significant main effect, i.e. worse average performance among CPs (D = 4.46, df = 1, p = 0.035; β_CP_ = −0.40, HPDI_95%_ = [−0.95, 0.01]), but no significant difference in the influence of trial type or rotation between the two groups (LR-test of full against main effects model: D = 0.16, df = 2, p = 0.92). Thus, the increased miss rate observed for CPs in recognizing faces was specific to faces and not the result of a general preference towards classifying test stimuli as unknown.

### Bielefeld Famous Face Test

**Figure 2:**
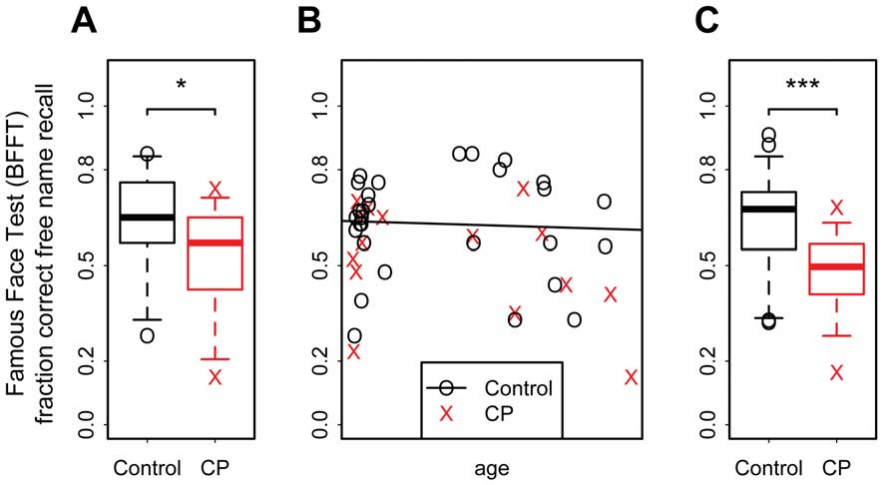
Bielefeld Famous Face Test. (A) Compared to controls, CPs showed worse recognition performance in free name recall. (B) Performance is influenced by confounding factors, e.g. age. (C) After accounting for confounding factors, performance differences between CPs and controls are more pronounced.

On average, participants with CP performed worse than controls in the recognition of famous faces. This decrease in performance can be partly explained by a lower benefit of increased media consumption in CPs compared to controls. Correcting individuals' performance for differences in age, gender, and media consumption reduces the variability and improves the separation between CPs and controls in comparison to the differences in raw performance (figure 2).

A direct comparison of raw performance revealed a lower percentage of correct free name recall in CPs compared to controls (Wilcoxon rank sum, W = 299.5, n_0_ = 29, n_CP_ = 15, p = 0.02 one-sided, see figure 2 A). We fitted a logistic regression model to account for confounding factors age (see figure 2 B), gender, and media consumption (print and TV). Accounting for these effects by comparing performance residuals increases the separation between CPs and controls (differences in residuals: W = 346, n_0_ = 29, n_CP_ = 15, p<0.001 one-sided, see figure 2 C).

We used model based comparisons to investigate group differences in the influence of the confounding factors. Firstly, we observed a significant main effect of group differences, with CPs performing worse than controls (LR-test of main effect against nullmodel, D = 46.89, df = 1, p<0.001; β_CP_ = −0.67, HPDI_95%_ = [−1.06, −0.27]). Secondly, we found significant interactions of participant group and media consumption (LR-test of full against main effect model, D = 10.71, df = 2, p = 0.004). Specifically, the positive effect of both types of media consumption observed for control participants was weaker for CPs: both for TV consumption (β_CP*TV_ = −0.12, CI_95%_ = [−0.32, 0.08]) as well as for print media consumption (β_CP*print_ = −0.30, CI_95%_ = [−0.52, −0.08]).

### Comparison of long-term recognition memory and Bielefeld Famous Face Test

**Figure 3:**
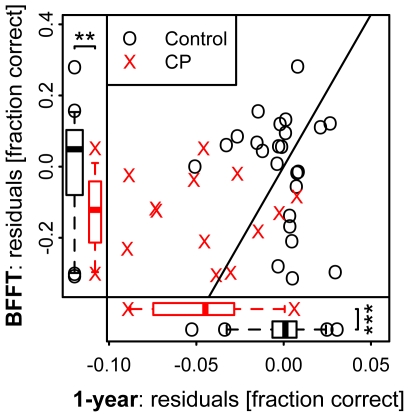
Comparison of famous face test (BFFT) and one-year recognition memory. Overall differences between CPs (X) and controls (O) are more pronounced in the test of one-year recognition memory than in the BFFT. Among controls the test results seem dissociated, with participants scoring bad in either of the two tests but not in both. Several controls who scored low in the BFFT, but all of them show normal performance in the standardized one-year recognition memory test.

For 37 participants (13 CPs and 24 controls) we obtained test results for both the Bielefeld Famous Face Test (BFFT) and the one-year recognition memory test. Comparing group differences in residuals between both tests, we observed that CPs' recognition difficulties were more pronounced in the controlled setting of the one-year recognition memory test (W = 273, p<0.001 one-sided) than in the famous face test (W = 240, p = 0.003 one-sided). Comparing the test results at an individual level, 8 out of 13 CPs tested showed significant deficits in the one-year recognition memory test, compared to 3 out of 15 in the famous face test. Overall, for nine CPs the deficit was larger in the one-year test (figure 3, values above the diagonal), and only for four was it larger in the BFFT.

Analysis of control participant's performance in both tests reveals a dissociation: Controls either score bad in one or the other test of long-term recognition but not in both (see figure 3). More specifically, all controls with a below average performance in the recognition of famous faces show a normal or average performance in our one-year recognition memory test.

### Intra- and inter-group variability across experiments of long- and short-term recognition tests

**Figure 4:**
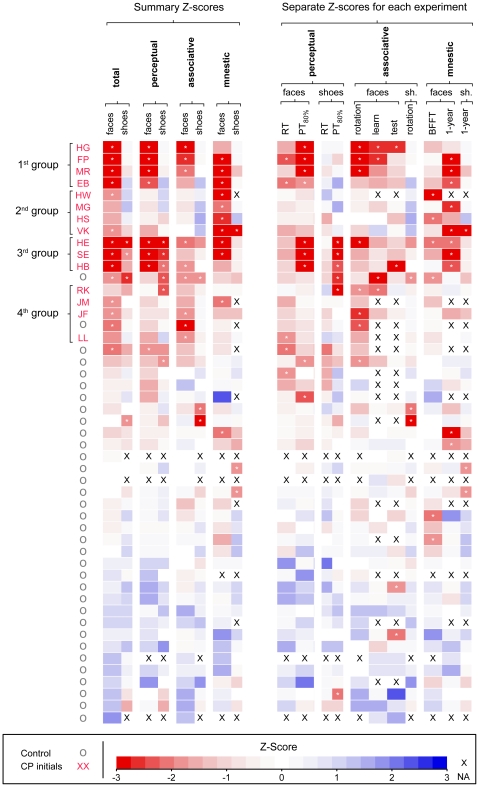
Overview of standardized test performance. Clustering participants according to their standardized test performance distinguishes CPs (top rows, marked by initials) from controls (bottom rows, marked by small circles) and reveals a dissociation of the deficits in CP into three homogeneous subtypes. In the table individual z-scores (color code at the bottom) are displayed for all of the tests (columns) based on estimated residuals for each participant. Significant deficits (modified t-test, 5% cutoff) are marked by an asterisk, missing values are denoted by X. Columns on the left side (Summary Z-scores) display aggregate performance across all or several tests, columns on on the right side display performance in individual tests (Separate Z-scores). Rows are ordered according to an unsupervised clustering on aggregate test results (complete linkage analysis). Abbreviations: RT = reaction time, PT_80%_ = presentation time needed for 80% correct performance, BFFT = Bielefeld famous face test.

Among the group of CPs, performance is quite variable across the different experimental categories (see figure 4). Looking only at significant deviations from control performance (marked by a small asterisk in figure 4), all of the 15 CPs had deficits in at least one of the individual tests of face recognition, 14 expressed a deficit in at least one of the three test categories of different aspects, and 11 showed a deficit in overall face recognition performance.

The differences in CPs' performance patterns across different tests point to three separate groups of prosopagnostic deficits:

The first group (HG, FP, MR, EB) is characterized by consistent perceptual, associative, and mnestic difficulties. The difficulties are selective to faces and don't extend to the recognition of shoes; they are already present at a perceptual level and propagate into associative and mnestic deficits, which leads to low total scores for faces (leftmost column).

Participants belonging to the second group (HW, MG, HS, VK) show clear deficits in long-term recognition memory, but don't exhibit perceptual or associative deficits in either face or shoe recognition. The deficits in long-term recognition can be more severe than among members of the first group with perceptual difficulties. In one case (VK) the mnestic deficit extends to shoe stimuli. As the deficit is restricted to mnestic aspects, only two of the four evince a deficit in overall face recognition performance.

Performance in the third group (HE, SE, HB) is characterized by simultaneous deficits in face and shoe recognition. The deficits are more pronounced in tests of perceptual aspects but can also extend to deficits of an associative and/or amnestic type.

The remaining four CPs (RK, JM,JF, and LL) showed mild and rather diffuse deficits in face and shoe recognition. While for RK the deficits seem to be of a more general nature extending to shoe recognition, the other three only show deficits in the recognition of faces.

**Figure 5:**
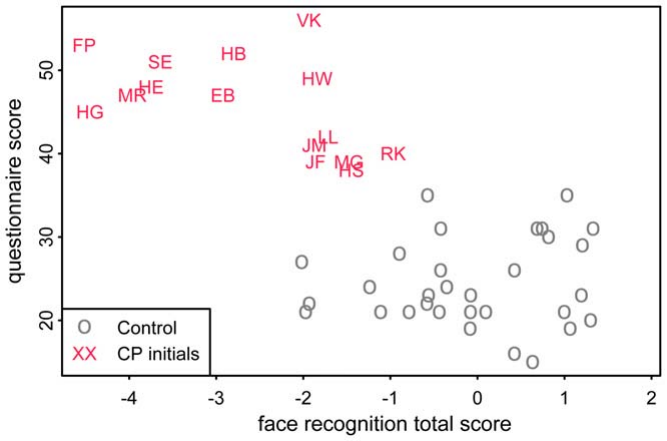
Correlation of experimentally tested deficits and self-assessment. Scores in questionnaire based self-assessment and overall residuals in experimental tests of face recognition performance are correlated in CPs (ρ = −0.55, p = 0.0345).

In general, there was good agreement between participants' self-assessment (questionnaire score) and face recognition performance (see figure 5), with a significant correlation (ρ = −0.55, p = 0.0345) across CPs.

## Discussion

### Summary

The aim of this study was to provide a characterization of long-term and short-term face recognition deficits in congenital prosopagnosia.

First, we assessed deficits in long-term recognition memory for faces and objects in a controlled test with a retention interval of one year. Out of the eleven CPs that participated in this experiment, eight showed clear deficits in recognizing faces (FP, MR, EB, MG, VK, HE, SE, HB), and an additional two showed performance clearly below the control average (HS, JF). This decrease in face recognition performance was mainly due to an increased miss rate among CPs. With the exception of one CP participant (VK) the deficit was selective to faces and didn't extend to recognizing individual non-face objects. In addition to this controlled test, we conducted a famous face test as it is commonly used to assess long-term recognition memory. In the famous face test only four of the CPs (HW, HS, HE) performed significantly below control average. A more detailed analysis of differences in performance in famous face recognition between controls and CPs revealed a decrease in the positive influence of media consumption (i.e. prior exposure or training).

Combining the results of long-term memory with prior results on perceptual and associative aspects of face and object recognition [44] allowed a more comprehensive characterization of CP. Overall, individuals in the CP group displayed a large heterogeneity in test performance, which is in line with previous studies [35], [54]. However, the observed heterogeneity is different from unstructured random variability but aligns with the separation of acquired prosopagnosia into distinct subtypes: apperceptive, associative, or amnestic.

Out of the 15 participants with CP tested in this study, seven show perceptual deficits in face processing (HG, FP, MR, EB, HE, SE, HB). In all of the seven cases the deficits extend to associative and/or mnestic aspects of face recognition. Four of the CPs only show mnestic deficits (HW, MG, HS, VK). The remaining four CPs evinced a more diffuse pattern of deficits. Although these cases don't fall into one of the three subtypes, three of them (JM, JF, LL) display an overall face recognition performance clearly below control average. Testing recognition performance for individual objects we found deficits of an apperceptive type in four CPs (HE,SE,HB,RK) and deficits of an amnestic type in one CP (VK).

Comparing the experimental results with a questionnaire based self-assessment, we found that all of the CPs tested in this study have provided more or less accurate estimates of the severity of their deficit. The only case with a strong deviation between self-assessment and behavioral measurements is VK, whose deficit is of an amnestic type and extends to the recognition of individual objects.

### Long-term recognition memory in congenital prosopagnosia

So far, experiments of long-term recognition memory in CP have been mostly restricted to tests of familiar face recognition, e.g. using pictures of family members [55], or tests of famous face recognition. Tests of familiar face recognition are necessarily adapted to each specific case, which renders comparisons of test results across different CP cases difficult. Tests of famous face recognition assume an apriori familiarization of participants with the faces through publicly available images and videos. The degree of familiarization depends to a large extent on each participants' social interest in the famous persons tested. Here, we used publicly available images of famous persons and found a strong influence of the degree of media consumption on recognition performance. This influence was also present among CPs, although to a lesser extent. Many CPs showed a normal recognition performance, which might be due to the presence of non-facial cues (hair, clothing, …) in the test images used.

In this study, we also present results on a controlled assessment of long-term recognition memory in CP for both faces and individual objects. The setup chosen provided the same degree of familiarization for each participant. We found that under this condition of equal familiarization long-term face recognition deficits in CP were more pronounced than in a test of famous face recognition. Moreover, by conducting an additional experiment with the same setup but using individual non-face object stimuli (Nike sneakers) we could rule out more general amnestic deficits unrelated to face processing.

In real life situations, the recognition of another person is often a mutual process: both persons simultaneously try to recognize each other and tend to communicate their results to the counterpart, e.g. by greetings or changes in mimic. As a positive identification signal can be given by any of the two persons involved, a compensatory, evasive strategy for CPs would be to simply wait until such a signal is given by the opponent, and later on explain the delayed response by inattentiveness (28). In an experimental setting, this compensatory strategy would lead to more misses (false negatives) than false positives, which was what we observed in the controlled one-year recognition memory experiment.

### A characterization of processing deficits

In acquired prosopagnosia, behavioral heterogeneity has been mostly explained by differences in the extent and location of the brain damage causing the deficit [5]–[7], [49]. In contrast, the deficit in CP is manifested as an endpoint of an inborn (endogenic), selective developmental impairment. Thus, while AP is caused by damaging a mature, functional system of face recognition, individuals with CP never evolve a fully functional face recognition system in the first place. Their deficit has to be interpreted in the context of a different developmental trajectory [56] into a mature but dysfunctional system.

Under normal developmental conditions, rudimentary abilities to discriminate between individuals can be observed already after the first days of age [57], [58] and continue to develop rapidly [59], [60]. Irrespective of the exact developmental processes underlying the subsequent functional specialization of cortical regions into a mature neural system for face recognition [61]–[63], this specialization presumably leads to an alignment between cortical location and functional process [52], [64]–[66]. Damage inflicted to a specific region can therefore lead to restricted deficits conditional on the interconnectedness and interdependence of the distributed processing [5]–[7], [49]. The spatial localization of specific functions in the ventral occipitotemporal cortex has enabled a characterization of processing deficits in AP based on the extent of the lesioned cortical regions.

Following the definitions proposed by Lissauer [2] AP has been divided into three subtypes:

- apperceptive - caused by a dysfunctional perceptual encoding,
- associative - resulting from deficits in the association of encoded percepts with individual objects, and
- amnestic - the deficit is restricted to accessing semantic information for known objects,

see also Damasio et al. [5] who use slightly different denominations. This symptomatic categorization of deficits parallels a functional modularization proposed in conceptual models of intact face recognition [50]–[53], and it aligns with the location of underlying cortical lesions roughly along a caudal-rostral axis [5], [6], [49].

In contrast, a similar categorization of CP into subtypes based on neurophysiological differences has remained elusive so far. First indications of neuroanatomical differences point to a volumetric reduction of the anterior fusiform gyrus [67], a region involved in more associative and mnestic aspects of face recognition, and a reduced structural connectivity in the ventral occipito-temporal cortex [68], a region involved in more apperceptive aspects of face recognition. Contrasting CPs and controls, Garrido et al. [43] found a positive correlation between face identification performance and gray matter volume in the left superior temporal sulcus and right fusiform gyrus, and a negative correlation between object recognition and volume in the lateral occipital cortex. In a large sample study, Dinkelacker et al. [69] found widespread areas of diminished gray matter density in the bilateral lingual gyrus, correlated with face memory success, as well as in the the right middle temporal gyrus and the dorsolateral prefrontal cortex. Irrespective of possible structural differences, findings of differences in functional MRI activations in core regions of the face processing system have been mixed, both using classical localizer paradigms [25], [54], [70] as well as adaptation paradigms [54], [71], [72].

In the following, we discuss the phenotypical heterogeneity observed in this study in three different contexts. The proposed characterization of CP subtypes is, firstly, interpreted with respect to cognitive and computational models of face recognition and, secondly, contrasted with possible underlying differences in the neuroanatomical structure. Thirdly, behavioral heterogeneity is discussed in the more general context of compensatory processing and evasive coping strategies.

#### Apperceptive prosopagnosia

Apperceptive prosopagnosia refers to a deficit in the early perceptual encoding of face images [5], [6], [49]. During perceptual encoding visual information is extracted from different locations with a certain efficiency, and the total information is obtained by integrating spatially across different locations and temporally across inspection time (cf. featural and holistic processing, see below). Deficits in perceptual encoding can occur either at the level of local efficiency or at the level of spatial integration. The process of perceptual encoding proceeds more slowly over time and/or reaches a saturation level that is too low for successful recognition. Thus, it was assumed, that in order to encode a sufficient amount of information, CPs need to inspect images longer, either due to a decrease in encoding efficiency or because they engage in a deliberate, series of attentional or fixational shifts to extract information at different spatial locations [44]. This hypothesis of longer inspection times in CP is consistent with previous studies of an increase in reaction times [27], [33], and reports of a more pronounced deficit under tachystocopic presentation in acquired prosopagnosia [73], [74], dispersed gaze behavior in CP [42], and a face specific increase in the recruitment of frontal-areas in CPs compared to controls [54]. Here, we assessed purely perceptual aspects in a same-view recognition task using frontal images by measuring reaction times for correct responses and presentation times needed to perform at an 80% correct level. Apperceptive deficits in face recognition were clearly present for seven CPs. For three of these seven, deficits extended to shoe recognition, and for one participant with CP the deficits were present only for shoe recognition.

In acquired prosopagnosia, apperceptive deficits are usually associated with damage to superior and inferior parts of the right posterior visual association areas [5]. It is assumed that under normal circumstances face-responsive regions in the inferior occipital gyrus are responsible for perceptual encoding and provide input to later areas of the core system of face processing [52]. Damage to early visual areas often also induces non-face related processing deficits, specifically deficits in within-object spatial coding after lesions to ventral occipito-temporal areas [17]. This decreased selectivity of apperceptive deficits was also observed in this study: The two CPs with clear overall deficits in object recognition (HE, SE) were cases of an apperceptive prosopagnosia, and conversely out of the seven apperceptive CPs, three also had apperceptive deficits with object recognition.

#### Associative prosopagnosia

In contrast to a purely perceptual deficit in perceptual encoding, associative deficits are characterized by a dysfunctional association of encoded percept and facial identity [5], [6], [49]. Under normal circumstances the information about the uniqueness of a face image, that is extracted during perceptual encoding, is associated with a specific facial identity such that future encounters of the same face lead to a recognition of this identity. To distinguish associative from perceptual deficits, that already occur at the level of matching identical images, we assessed recognition accuracy in a delayed recognition task using rotated and differently illuminated test images. In previous studies using rotated [27], or rotated, differently illuminated, and noised stimuli [40], CPs consistently evinced worse performance than controls. In this study, we observed associative deficits in face recognition for seven CPs, and borderline performance in four CPs.

However, as already noted by Lissauer [2] the distinction between associative and apperceptive types of agnosia is anything but clear; he suspected that all observable cases of prosopagnosia would be rather a mixture between the two extremes. Comparing apperceptive and associative deficits, we found that all CPs with an apperceptive deficit also show deficits or below average performance in association. The reverse doesn't hold, as two CPs (LL, JF) show associative deficits but average performance w.r.t. perceptual aspects. This pattern of perceptual deficits leading to associative deficits aligns with models of hierarchical information processing [50]–[53]: Dysfunctional processing at lower areas can evince deficits in functions that are normally associated with processing in higher areas.

Based on a classic model of face recognition and prosopagnosia [50]. Young and Burton [75] simulated associative prosopagnosia as a disconnection between face recognition units, where the encoded percept or facial memory is stored, and personal identity nodes. According to this model, if early perceptual encoding is performed in inferior occipital regions, e.g. the occipital and the fusiform face area (OFA and FFA), and facial memories are stored in anterior temporal regions, associative prosopagnosia might be caused by a disconnection of the tracts connecting the posterior occipitotemporal regions with more anterior temporal regions, e.g. the inferior longitudinal fasciculus (ILF). A contrary view posits that facial memories are already stored in the FFA and associative prosopagnosia results from a disconnection between OFA and FFA (see [49], for a discussion). Recent studies on brain abnormalities in CP have observed a reduction in the structural connectivity of ventral visual areas that is most prominent in the right ILF [68], and a decrease in grey matter volume in the right fusiform and inferior temporal gyri [43]. In both studies the reductions were correlated in magnitude with deficits in face recognition performance.

#### Amnestic prosopagnosia

Amnestic prosopagnosia is associated with deficits in establishing and maintaining the long-term stability of an association between a facial identity and a semantic identity [5], [49]. In principle, amnestic deficits can occur in the presence of facial identities. For example, one could recognize a face as a familiar face, without being able to access further semantic information regarding the bearer of the face. Here, amnestic deficits were assessed by the two tests of long-term recognition memory outlined above. In the BFFT the semantic information requested was the name of the famous person depicted; in the one-year recognition memory test we asked whether a face belonged to the group of four target faces introduced one year before. In both cases simple familiarity with a face doesn't enable a correct answer, although familiarity might indirectly ease the access to semantic information. For example, one could make a guess at the profession of a familiar person by factoring in one's own interests: “I'm not interested in sports, but very interested in politics. Therefore, if I know this woman, then she is more likely to be a politician than a sports player.” Based on these two tests we observed amnestic deficits in face recognition for ten CPs, and borderline performance in two CPs. For one participant with CP (VK) the deficit in long-term recognition memory was not specific to faces. In agreement with hierarchical processing of facial information, we found that all cases of apperceptive CPs show deficits or borderline performance in tests of amnestic aspects. In contrast, the two cases with pure associative deficits (LL, JF) perform normal.

Amnestic deficits in acquired prosopagnosia have mostly been associated with damage to anterior temporal regions [5]. In CP amnestic deficits could be caused by an insufficient “storage” capacity in areas associating faces with semantic information. In artificial neural networks a decrease in capacity can be caused by a decrease in the number of neurons. Taking cortical volume as an estimate of the number of neurons, this view is consistent with findings of a volumetric reduction in the anterior fusiform gyrus in CP cases which is correlated in size with performance in a famous face test [67].

### Diagnostic assessment

In this study, diagnosis of CP was based on a semi-structured interview which involves subjective reports on perceived face recognition difficulties, such as a reported uncertainty in face recognition, prolonged recognition times surpassing socially accepted time spans, and the development of compensatory strategies. Relying on a structured but subjective assessment of real life difficulties instead of a more controlled assessment of face recognition abilities under experimental settings, has benefits as well as caveats. Previous investigations have criticized a reliance on self-assessment [23] and often adopted additional, conservative inclusion criteria based on significant deficits in experimental tests [27], [76]. The reverse position, that experimental tests of face recognition might not be suited to accurately reflect the complexity of the processes underlying face recognition in real life situations, has only been given scarce notice. For example, evaluating two previously often used tests of unfamiliar face recognition, Duchaine and Weidenfeld [77] found that normal scores on these tests are not indicative of normal face recognition.

In this study, we found a clear correlation between the self-reported difficulties of CPs, measured using a standardized questionnaire [33], and their overall deficits across all of the experimental tests applied. Although the correlation was far from perfect, this demonstrates that CPs, on the one hand, are able to report more or less accurately on the extent of their deficits and that, on the other hand, an extensive formal assessment, including tests of long-term recognition memory, can capture the intricacies of real life face recognition.

In contrast to a self-assessment of their own deficits, CPs are often unaware of affected family members. For example, in a collection of developmental prosopagnosics, 11 out of 19 questioned reported affected relatives, whereas the others were unsure or exclude other impaired family members [78]. As far as we understood, these data are compiled by asking the index CPs for their family history and not by studying each family member individually. In this and previous studies, whenever we explicitly tested the family members of a CP participant personally one by one, we found affected relatives in almost all cases [32]. We therefore coined the term hereditary prosopagnosia (HPA).

## Methods

The experiments were conducted at different times and locations. Experiments with CP participants (CPs) took place at the Institut für Humangenetik, Westfälische-Wilhelms-Universität, Münster, experiments with control participants took place at the Max Planck Institute for Mathematics in the Sciences, Leipzig. The famous face test was conducted at the end of 2006, the test of long-term recognition memory one year later at the end of 2007.

### Participants

As a total across all experiments, we initially tested 16 CPs and 36 age-matched controls. One of the CPs tested (MB) didn't show face recognition impairments in any of the experimental tests, although reporting difficulties in real-life situations. MB has a strabismus convergens, on which she was operated on three times during childhood. However, she still reported on perceiving diplopic images and difficulties with stereopsy. To avoid a possible bias (see also discussion) we excluded MB, as well as the corresponding two age-matched controls, from all further analysis. This exclusion lead to a final total of 15 CPs and 34 controls.

Participants' age at first testing, i.e. end of 2006, varied between 20 and 68 years, with a mean age of 37.3 years (sd: 17.9) for CPs and 37.7 years (sd: 16.8) for controls.

All 15 CPs as well as 29 controls participated in the Bielefeld famous face test. In addition, all of these 44 participants were made familiar with four target faces and four target shoes in a series of experiments on short-term recognition (see below), and 13 CPs and 25 controls were able to participate one year later in the test of long-term recognition memory. Due to these differences in participation between the two tests, age-matching was less stringent in the long-term recognition memory test where participating CPs were on average older than control participants (mean age of 34.1 and 39.5 for controls and CPs respectively).

For an overview on individual participation in specific experiments see also Figure 4.

#### Ethics statement

All CPs and controls provided written informed consent before participation. The study was approved by the ethical committee of the University of Muenster, Germany, protocol No 3XKenn2.

#### Participants with congenital prosopagnosia

Diagnosis of CP was based on a semi-structured interview which includes questions on everyday-problems with face and object recognition, mental imagery and avoidance strategies (see below). Overall, most CPs had normal or borderline normal basic-level object recognition abilities as measured by BORB tests 6,7,10,13 [79] and VOSP tests 2,4,6 [80]. Only one, HW, had consistent deficits across three tests (BORB 10A hard; VOSP 2,4).

A neurological exam of each participant indicated normal clinical status. We explicitly asked for neurological and psychiatric disorders in the family. In all cases pregnancy was uneventful and no complications such as perinatal asphyxia were reported. No participant was aware of any traumatic, comatose event or infectious disease (encephalitis or meningitis) during childhood. No CT scan, MRI of the head or EEG was performed which might retrospectively suggestive of an neurological disorder or atypical neurological reaction (migraine, epilepsy). Furthermore there were no hints of any delusional symptoms or autism spectrum disorder.

#### Diagnostic interview

Diagnosis of prosopagnosia was made by a semi-structured interview of about 90 minutes [28]–[32], [81]. In order to be diagnosed with CP, participants had to meet the following criteria:

- Uncertainty in face recognition: Not recognizing familiar people unexpectedly or in crowded places, confusing unknown persons with familiar persons. Only anecdotal mentioning of not recognizing people is not taken as a positive criterion.
- Prolonged recognition time for faces (in terms of a socially accepted span of time).
- Development of compensatory strategies as sign of a longstanding frequent problem. Strategies can include either adaptive behavior (identification by e.g. voice, gait, clothing) or avoidance behavior (e.g. looking absent-minded, cancel meetings).
- Surprising anecdotal stories (problems in following actors in a movie).

In addition, a family history of at least one affected first degree relative renders an hereditary origin of the difficulties more likely, thereby increasing the probability of congenital prosopagnosia - including hereditary prosopagnosia.

#### Screening questionnaire

All participants completed a screening questionnaire which consists of 15 questions on a five-point rating scale [31]. Three dummy questions not specific for prosopagnosia were also included. The three questions consisted of: whether one can distinguish male and female faces, whether one can say that a face is attractive and whether one can read emotions. Each question was scored individually with 1 to 5 points, where larger scores indicate noticeable difficulties, resulting in a total of 15 to 75 points.

### Experiments

#### Long-term recognition memory test

The long-term recognition memory made use of the familiarization of participants with different face and object stimuli established a year earlier in a series of experiments on short-term recognition memory (see below). It assessed whether participants were still able to judge whether a stimulus was among the selected four target stimuli learned previously. In each experiment, a total of 20 face (or shoe) stimuli was shown: Four target stimuli (two male, two female) and 16 distractor stimuli (eight male, eight female) which were all presented one year earlier.

Familiarization during these prior experiments consisted of at least four presentations of the target stimuli in frontal view for an unlimited duration, four sessions of feedback-training with two trials for each target stimulus and one for each distractor stimulus in each session, and two test sessions without feedback and limited presentation times with a total of 22 frontal and 12 rotated presentations for each target face (24 and 8 for each target shoe, respectively). As participants were familiar with both target as well as distractor stimuli, correct judgments couldn't have been made based on familiarity alone. Matched controls had exactly the same experimental setup as their respective CPs.

Both experiments were performed in two parts of 160 presentations each: 8 repetitions of 20 stimuli, 4 targets, 16 distractors, in randomized order. In the first part participants were presented with rotated target and distractor stimuli (±30° for faces and side/top-view for shoes), in the second part with non-rotated images (frontal and oblique resp.). In addition, target face stimuli were shown in one of four illumination conditions, where only one illumination condition was the same as the illumination used previously. Stimuli were presented until participants responded and were separated by a blank screen presented for one frame.

The face stimuli were obtained from the publicly available Face Database of the MPI for Biological Cybernetics (see [82], for details on the database creation) which contains snapshots of 3D-scans of 200 heads of caucasian people (without hair) taken at seven rotations (frontal view and 3 rotations in each direction of 30°, 60° and 90°). These snapshots were used as distractor stimuli. Target face stimuli were generated using the four individual full head models in the Face Database (two male and two female heads). Snapshots of the full head models under the same rotations (30°, 60° and 90°) were generated using Blender free open source 3D content creation suite (http://www.blender.org, open-source). All snapshots are 8-bit color images of 256×256 pixels.

The shoe stimuli were obtained as snapshots of different sneakers obtained from http://nikeid.nike.com. A total of 53 distractor shoes and 4 target shoes were used, all under the available three different rotation conditions (oblique, side and top view).

All images were presented on a IIYAMA Vision Master Pro514 monitor (22′, at 200 Hz) with a resolution of 800×600 and images subtended 130 pixels×190 pixels, i.e. 65 mm×85 mm or 3.5°×4.3° at the initial seating distance of 1 m.

The experiment was run using the open-source flashdot experimental psychophysics presentation software [83] which is available at http://www.flashdot.info.

#### Bielefeld Famous Face Test

The Bielefeld Famous Face Test (BFFT) was originally developed as a test for antero- and posterograde amnesia [47], [48]. It includes grayscale portrait photographs, including non-facial cues, of people famous in Germany which were taken in different decades and collected from publicly available sources. Persons depicted include pictures of globally famous persons of non-German, e.g. Hillary Clinton (n = 10), and German origin, e.g. Boris Becker (n = 14), as well as persons famous in Germany but not widely known outside of Germany, e.g. Marcel Reich-Ranicki (n = 16). Here, we only included pictures taken after the German reunification in 1990 and only tested for differences in free name recall. Images were printed out and presented sequentially to the participants who were free to take as much time to respond as they wished.

#### Short-term recognition memory tests

In total, we conducted eight experiments testing apperceptive and associative aspects of face and object recognition with short retention intervals (see [44] for the original data and a detailed report on the short-term recognition memory tests applied).

In the experiments testing perceptual aspects, a standard setting was used to assess recognition of frontal images of faces and shoes. Participants were familiarized with four individual target stimuli (identical to those later used for the long-term recognition memory test) and later on had to identify the targets amongst a group of distractor stimuli in a two-alternative forced choice paradigm (target vs. non-target). We focused our investigation on whether longer reaction times can be attributed to longer inspection of the images or a longer decisional component. First, we measured participants' reaction times under the condition of unlimited presentation (later referred to as RT -faces/shoes ). Second, we used an adaptive sampling strategy to estimate the presentation time at which a participant performs with an accuracy of 80% (PT_80%_ - faces/shoes).

The first experiment assessing associative aspects tested participants' ability to generalize from the learned frontal view to a novel view of the stimulus (rotation - faces/shoes). While recognition of stimuli taken under identical viewing conditions can be solved by image matching, rotation in depth, which occurs frequently under natural viewing conditions, at least diminishes the applicability of similar compensatory strategies. In order to isolate the influence of rotation and to avoid statistical ceiling (or floor) effects in the performance, for each CP and his/her respective matched controls, rotated images were displayed at a presentation time at which the CP participants had previously achieved an accuracy of 90% in the recognition of frontal views – estimated using the data obtained during the adaptive sampling experiment described above.

In two further experiments on associative aspects, we studied generalization across small changes in viewpoint (±5° in-depth rotation around vertical and horizontal axis, different illumination) while keeping presentation times fixed at either 50 ms, 150 ms, 450 ms, or 750 ms. This limitation in presentation times was imposed either during the encoding, i.e. learning, of a novel face (learn - faces) or during the decoding, i.e. recognition, of a previously learned face (test – faces).

### Statistical analysis

To assess whether individual CPs showed an abnormal performance in any of the tests and to test for significance of differences in the influence of experimental variables between the control and the CP group, we used generalized linear mixed models (GLMMs, see e.g. [84], for an introduction).

#### Calculation of abnormality scores

To compare individuals' performance across different tests, we calculated standardized abnormality scores, based on GLMM nullmodels, that are corrected for differences in confounding factors (e.g. age).

Initially, for each test a nullmodel was selected using all control observations. After selection of a nullmodel, which specified the error type, link-function, fixed- and random-effects, we estimated the fixed-effect parameters using only the observations of controls, termed the control model.

This control model was then used to calculate residuals for each CP participant. The residual for the j-th participant, with observed outcome y_j_ and predictors (contributing factors) x_j_,, is defined as the difference between actual performance, y_i_, and expected performance under the control nullmodel, y(x_j_).

For control participants residuals were calculated similarly, this time using individualized control models. The individualized control model for the i-th control was obtained by estimating the parameter values of the fixed-effects in the nullmodel based on all control observations except those of individual i. Roughly speaking, this additional step of calculating controls' residuals based on individualized control models reduced the risk of fitting model parameters too closely to the control data, thereby modeling the idiosyncrasies of each individuals' performance and underestimating the variability in control performance. By using this “leave-one-out” estimation of expected control performance one obtains an unbiased estimate of the variance in control residuals, i.e. a leave-one-out cross-validation estimate [85].

Finally, all residuals are then transformed into z-scores by subtracting control mean and dividing by the standard deviation of control residuals.

To highlight patterns in CPs' deficits, we calculated average scores for each of the three test categories (perceptual, associative, mnestic), as well as an overall score; the individual z-scores in the corresponding tests were averaged for each individual, and afterwards again transformed into z-scores based on control standard deviations. If a participant's score falls below the 5% quantile of the corresponding t-distributon [86], the performance will be judged abnormal and it will be referred to as a deficit.

The proposed calculation of abnormality scores deviates from those proposed by Crawford and Garthwaite [87], [88] in two aspects: On the one hand, it is more general, as it extends the case of linear regression models to generalized linear mixed models. On the other hand, here the unconditional variance of control residuals is used, whereas Crawford and Garthwaite [87], [88] calculate the residual variance conditional on the observed value of the confounding factors. Conditioning on the confounding variables accounts for an increase in residual variance that is due to possible errors in the estimation of model parameters, i.e. the estimate of residual variance will increase in magnitude the further the values of the confounding factors are from the control mean. In this study, the primary focus was to provide a comparison of individual's performance across different tests in order to reveal patterns of correlated deficits; the estimation of exact abnormality scores for each individual was only of secondary importance. Therefore, we chose to enlarge the range of possible models to include generalized linear mixed models, at the expense of possibly slightly exaggerated abnormality scores for CPs and(!) controls with “abnormal” values of the confounding factors.

#### Model based comparisons

First, a nullmodel that always included fixed effects for age and all experimental variables (e.g. presentation time) as well as random effects that allow for individual variation in the mean and in the influence of experimental variables was fitted. Based on this nullmodel, alternative, nested models were constructed by subsequently adding group differences in the influence of fixed effects, i.e. firstly a mean difference between the groups (main effect), secondly an interaction of group and experimental variables (first-order effects), and analogously for higher order interactions. Comparison of nested models was based on differences in the log-likelihood of the models, i.e. a likelihood ratio test (LR-test). In cases of significant differences we calculated Bayesian maximum posterior estimates as well as highest posterior density intervals with 95% support (HPDI_95%_) for the interaction effects.

#### Description of the GLMMs used

In the analysis of the famous face test, the nullmodel was fitted as a a binomial GLMM nullmodel with logit-link (logistic regression model) which included fixed effects of age, gender, and both TV and print media consumption (both discretized as <1 h, 1–2 h, 3–7 h, >7 h per week).

In the analysis of the long-term recognition experiments, we again used a binomial GLMM nullmodel with logit-link including age, trial type (target present or not present) and rotation (frontal or rotated) as fixed effects and participant identity as a random effect. In the shoes experiment, the influence of trial type varied significantly across participants and was thus included as an additional random effect. Group differences in the influence of fixed effects were tested for a combined influence of trial type and rotation (full model).

All of the GLMM nullmodels used to analyze the experiments on short-term recognition also included fixed effect of age. The type of error distribution, choice of link-function, fixed- and random-effects differed between model, see [44] for details.

#### Statistical software

All data analysis and statistical testing was conducted using the statistical programming language R [89]. Fitting of generalized linear mixed models (GLMMs) was done using the R packages lme4 [90] and MCMCglmm [91]. The algorithms used in lme4, as well as the model based comparisons conducted here, are described by the main contributor to the lme4 package in more detail in [92]. To test for significant differences likelihood ratio tests were performed where we assumed a χ2 distribution of the test statistics with degrees of freedom equal to the difference in the number of parameters. In testing significance of fixed effects in mixed models, the χ^2^ approximation tends to produce p-values that are too small [92]. Hence, if the selected model included interaction effects, the model was again fit with MCMCglmm to obtain Bayesian maximum posterior estimates (β) and highest posterior density intervals with 95% support (HPDI_95%_) for parameter estimates of interaction effects [93]. As prior distributions for the Bayesian model fitting we used a multivariate normal distribution with zero mean and a diagonal covariance matrix with large variances (σ = 10^10^) for fixed effects and an inverse Wishart distribution with degrees of freedom equal to one and the inverse scale equal to the unconditional variance of the response variable.

## References

[1] Bodamer J (1947). Die Prosop-Agnosie.. Arch Psych Nervenkr.

[2] Lissauer H (1890). Ein Fall von Seelenblindheit nebst einem Beitrag zur Theorie derselben.. Arch Psychiatr Nervenkr.

[3] Wilbrand H (1892). Ein Fall von Seelenblindheit und Hemianopsie mit Sectionsbefund.. Dtsch Z Nervenheilk.

[4] Hecaen H, Angelergues R (1962). Agnosia for faces (prosopagnosia).. Archives of neurology.

[5] Damasio A, Tranel D, Damasio H (1990). Face Agnosia and the Neural Substrates of Memory.. Annual Review of Neuroscience.

[6] De Renzi E, Faglioni P, Grossi D, Nichelli P (1991). Apperceptive and associative forms of prosopagnosia.. Cortex.

[7] De Renzi E, Perani D, Carlesimo G, Silveri M, Fazio F (1994). Prosopagnosia can be Associated with Damage Confined to the Right-Hemisphere - An MRI and PET Study and a Review of the Literature.. Neuropsychologia.

[8] Barton JJS, Cherkasova M, O'Connor M (2001). Covert recognition in acquired and developmental prosopagnosia.. Neurology.

[9] Quaglino A, Borelli GB, Della Sala S, Young AW (2003). Quaglino's 1867 case of prosopagnosia.. Cortex.

[10] Mazzucchi A, Biber C (1983). Is prosopagnosia more frequent in males than in females?. Cortex.

[11] Bauer R (1984). Autonomic Recognition of Names and Faces in Prosopagnosia - A Neuropsy-chological Application of the Guilty Knowledge Test.. Neuropsychologia.

[12] Tranel D, Damasio H, Damasio A (1995). Double Dissociation Betwen Overt and Covert Face Recognition.. Journal of Cognitive Neuroscience.

[13] Bobes MA, Lopera F, Garcia M, Díaz-Comas L, Galan L (2003). Covert matching of unfamiliar faces in a case of prosopagnosia: an ERP study.. Cortex.

[14] Sperber S, Spinnler H (2003). Covert person recognition: Its fadeout in a case of temporal lobe degeneration.. Cortex.

[15] Barton JJS, Press DZ, Keenan JP, O'Connor M (2002). Lesions of the fusiform face area impair perception of facial configuration in prosopagnosia.. Neurology.

[16] Barton JJS, Zhao J, Keenan JP (2003). Perception of global facial geometry in the inversion effect and prosopagnosia.. Neuropsychologia.

[17] Barton JJS, Cherkasova M (2005). Impaired spatial coding within objects but not between objects in prosopagnosia.. Neurology.

[18] Barton JJS, Cherkasova M (2003). Face imagery and its relation to perception and covert recognition in prosopagnosia.. Neurology.

[19] Humphreys K, Avidan G, Behrmann M (2007). A detailed investigation of facial expression processing in congenital prosopagnosia as compared to acquired prosopagnosia.. Experimental Brain Research.

[20] McNeil JE, Warrington EK (1993). Prosopagnosia: a face-specific disorder.. The Quarterly Journal of Experimental Psychology Section A.

[21] McConachie HR (1976). Developmental prosopagnosia. A single case report.. Cortex.

[22] Ariel R, Sadeh M (1996). Congenital visual agnosia and prosopagnosia in a child: a case report.. Cortex.

[23] De Haan E (1999). A familial factor in the development of face recognition deficits.. Journal of Clinical and Experimental Neuropsychology.

[24] Kress T, Daum I (2003). Developmental prosopagnosia: a review.. Behavioural neurology.

[25] Hasson U, Avidan G, Deouell LY, Bentin S, Malach R (2003). Face-selective activation in a congenital prosopagnosic subject.. Journal of Cognitive Neuroscience.

[26] Behrmann M, Avidan G (2005). Congenital prosopagnosia: face-blind from birth.. Trends in Cognitive Sciences.

[27] Behrmann M, Avidan G, Marotta J, Kimchi R (2005). Detailed exploration of face-related processing in congenital prosopagnosia: 1. Behavioral findings.. Journal of Cognitive Neuroscience.

[28] Kennerknecht I, Grueter T, Welling B, Wentzek S, Horst J (2006). First report of prevalence of non-syndromic hereditary prosopagnosia (HPA).. American Journal of Medical Genetics.

[29] Grueter M, Grueter T, Bell V, Horst J, Laskowski W (2007). Hereditary Prosopagnosia: the First Case Series.. Cortex.

[30] Kennerknecht I, Pluempe N, Edwards S, Raman R (2007). Hereditary prosopagnosia (HPA): the first report outside the Caucasian population.. Journal of Human Genetics.

[31] Kennerknecht I, Ho NY, Wong VCN (2008a). Prevalence of hereditary prosopagnosia (HPA) in Hong Kong Chinese population.. American Journal of Medical Genetics ,.

[32] Kennerknecht I, Pluempe N, Welling B (2008b). Congenital prosopagnosia a common hereditary cognitive dysfunction in humans.. Frontiers in Bioscience.

[33] Duchaine BC, Nakayama K (2005). Dissociations of Face and Object Recognition in Developmental Prosopagnosia.. Journal of Cognitive Neuroscience.

[34] Duchaine BC (2006). Prosopagnosia as an impairment to face-specific mechanisms: Elimination of the alternative hypotheses in a developmental case.. Cognitive Neuropsychology.

[35] Schmalzl L, Palermo R, Coltheart M (2008a). Cognitive heterogeneity in genetically based prosopagnosia: A family study.. Journal of Neuropsychology.

[36] Lange J, De Lussanet M, Kuhlmann S, Zimmermann A, Lappe M (2009). Impairments of Biological Motion Perception in Congenital Prosopagnosia.. PLoS ONE.

[37] Grueter T, Grueter M, Bell V, Carbon CC (2009). Visual mental imagery in congenital prosopagnosia.. Neuroscience Letters.

[38] Garrido L, Duchaine B, Nakayama K (2008). Face detection in normal and prosopagnosic individuals.. Journal of Neuropsychology.

[39] Le Grand R, Cooper PA, Mondloch CJ, Lewis TL, Sagiv N (2006). What aspects of face processing are impaired in developmental prosopagnosia?. Brain and Cognition.

[40] Duchaine BC (2000). Developmental prosopagnosia with normal configural processing.. Neuroreport.

[41] de Gelder B, Rouw R (2000). Configural face processes in acquired and developmental prosopagnosia: evidence for two separate face systems?. Neuroreport.

[42] Schwarzer G, Huber S, Grueter M, Grueter T, Gross C (2007). Gaze behaviour in hereditary prosopagnosia.. Psychological Research.

[43] Garrido L, Furl N, Draganski B, Weiskopf N, Stevens J (2009). Voxel-based morphometry reveals reduced grey matter volume in the temporal cortex of developmental prosopagnosics.. Brain.

[44] Stollhoff R, Jost J, Elze T, Kennerknecht I (2010). The Early Time Course of Compensatory Face Processing in Congenital Prosopagnosia.. PLoS ONE.

[45] Palmeri T, Gauthier I (2004). Visual object understanding.. Nature Reviews Neuroscience.

[46] Gauthier I, Tarr MJ, Moylan J, Skudlarski P, Gore JC (2000a). The fusiform “face area” is part of a network that processes faces at the individual level.. Journal of Cognitive Neuroscience.

[47] Jänicke C, Markowitsch HJ, Fast K (2001). Die Entwicklung des Bielefelder Famous Faces Test..

[48] Kalbe E, Brand M, Thiel A, Kessler J, Markowitsch HJ (2008). Neuropsychological and neural correlates of autobiographical deficits in a mother who killed her children.. Neurocase.

[49] Fox CJ, Iaria G, Barton JJS (2008). Disconnection in prosopagnosia and face processing.. Cortex.

[50] Bruce V, Young AW (1986). Understanding face recognition.. British Journal of Psychology.

[51] Breen N, Caine D, Coltheart M (2000). Models of face recognition and delusional misidentification: A critical review.. Cognitive Neuropsychology.

[52] Haxby JV, Hoffman E, Gobbini M (2000). The distributed human neural system for face perception.. Trends in Cognitive Sciences.

[53] Ellis HD, Lewis M (2001). Capgras delusion: a window on face recognition.. Trends in Cognitive Sciences.

[54] Avidan G, Hasson U, Malach R, Behrmann M (2005). Detailed exploration of face-related processing in congenital prosopagnosia: 2. Functional neuroimaging findings.. Journal of Cognitive Neuroscience.

[55] Schmalzl L, Palermo R, Green M, Brunsdon R, Coltheart M (2008b). Training of familiar face recognition and visual scan paths for faces in a child with congenital prosopagnosia.. Cognitive Neuropsychology.

[56] Thomas M, Karmiloff-Smith A (2003). Are developmental disorders like cases of adult brain damage? Implications from connectionist modelling.. Behavioral and Brain Sciences.

[57] Pascalis O, de Schonen S (1994). Recognition memory in 3- to 4-day-old human neonates.. Neuroreport.

[58] Turati C, Bulf H, Simion F (2008). Newborns' face recognition over changes in viewpoint.. Cognition.

[59] De Haan M, Johnson MH, Maurer D, Perrett D (2001). Recognition of individual faces and average face prototypes by 1-and 3-month-old infants.. Cognitive Development.

[60] Mondloch CJ, Geldart S, Maurer D, Le Grand R (2003). Developmental changes in face processing skills.. Journal of Experimental Child Psychology.

[61] De Haan M, Humphreys K, Johnson MH (2002). Developing a brain specialized for face perception: A converging methods approach.. Developmental Psychobiology.

[62] Scherf KS, Behrmann M, Humphreys K, Luna B (2007). Visual category-selectivity for faces, places and objects emerges along different developmental trajectories.. Developmental Science.

[63] Polk TA, Park J, Smith MR, Park DC (2007). Nature versus nurture in ven- tral visual cortex: A functional magnetic resonance Imaging study of twins.. Journal of Neuroscience.

[64] Kanwisher N, McDermott J, Chun MM (1997). The fusiform face area: a module in human extrastriate cortex specialized for face perception.. Journal of Neuroscience.

[65] Gauthier I, Tarr MJ, Moylan J, Skudlarski P, Gore JC (2000b). The fusiform “face area” is part of a network that processes faces at the individual level.. Journal of Cognitive Neuroscience.

[66] Hoffman E, Haxby JV (2000). Distinct representations of eye gaze and identity in the distributed human neural system for face perception.. Nature Neuroscience.

[67] Behrmann M, Avidan G, Gao F, Black S (2007). Structural imaging reveals anatomical alterations in inferotemporal cortex in congenital prosopagnosia.. Cerebral Cortex.

[68] Thomas C, Avidan G, Humphreys K, Jung KJ, Gao F (2009). Reduced structural connectivity in ventral visual cortex in congenital prosopagnosia.. Nature Neuroscience.

[69] Dinkelacker V, Grueter M, Klaver P, Grueter T, Specht K (2010). Congenital Prosopagnosia: Multistage Anatomical and Functional Deficits in Face Processing Circuitry.. Journal of Neurology.

[70] von Kriegstein K, Kleinschmidt A, Giraud AL (2006). Voice recognition and cross-modal responses to familiar speakers' voices in prosopagnosia.. Cerebral Cortex.

[71] Williams MA, Berberovic N, Mattingley JB (2007). Abnormal fMRI adaptation to unfamiliar faces in a case of developmental prosopamnesia.. Current biology.

[72] Avidan G, Behrmann M (2009). Functional MRI Reveals Compromised Neural Integrity of the Face Processing Network in Congenital Prosopagnosia.. Current biology.

[73] Faust C (1947). Partielle Seelenblindheit nach Occipitalhirnverletzung mit besonderer Beeintraechtigung des Physiognomieerkennens.. Der Nervenarzt.

[74] Gauthier I, Behrmann M, Tarr MJ (1999). Can face recognition really be dissociated from object recognition?. Journal of Cognitive Neuroscience.

[75] Young AW, Burton AM (1999). Simulating face recognition: Implications for modelling cognition.. Cognitive Neuropsychology.

[76] Duchaine BC, Nakayama K (2006). The Cambridge Face Memory Test: results for neurologically intact individuals and an investigation of its validity using inverted face stimuli and prosopagnosic participants.. Neuropsychologia.

[77] Duchaine BC, Weidenfeld A (2003). An evaluation of two commonly used tests of unfamiliar face recognition.. Neuropsychologia.

[78] Duchaine BC (2008). Comment on Prevalence of Hereditary Prosopagnosia (HPA) in Hong Kong Chinese Population.. American Journal of Medical Genetics.

[79] Riddoch JM, Humphreys GW (1993). BORB: Birmingham Object Recognition Battery.

[80] Warrington EK, James M (1991). The Visual Object and Space Perception Battery.

[81] Grueter M (2004). Genetik der kongenitalen Prosopagnosie [Genetics of congenital prosopagnosia]..

[82] Troje NF, Bülthoff HH (1996). Face recognition under varying poses: the role of texture and shape.. Vision Research.

[83] Elze T (2009). FlashDot - A platform independent experiment generator for visual psychophysics.. Journal of Vision.

[84] Tuerlinckx F, Rijmen F, Verbeke G, De Boeck P (2006). Statistical inference in generalized linear mixed models: a review.. The British journal of mathematical and statistical psychology.

[85] Efron B, Tibshirani R (1993). An introduction to the bootstrap.

[86] Crawford J, Howell D (1998). Comparing an individual's test score against norms derived from small samples.. Clinical Neuropsychology.

[87] Crawford J, Garthwaite P (2006). Comparing patients' predicted test scores from a regression equation with their obtained scores: A significance test and point estimate of abnormality with accompanying confidence limits.. Neuropsychology.

[88] Crawford J, Garthwaite P, Azzalini A, Howell D, Laws K (2006). Testing for a deficit in single-case studies: Effects of departures from normality.. Neuropsychologia.

[89] R Development Core Team (2009). R: A Language and Environment for Statistical Computing. R Foundation for Statistical Computing, Vienna, Austria..

[90] Bates D, Maechler M (2009). lme4: Linear mixed-effects models using S4 classes..

[91] Hadfield J (2009). MCMC methods for Multi-response Generalised Linear Mixed Models: The MCMCglmm R Package..

[92] Faraway JJ (2006). Extending the linear model with R: generalized linear, mixed effects and nonparametric regression models.

[93] Baayen R, Davidson D, Bates D (2008). Mixed-effects modeling with crossed random effects for subjects and items.. Journal of Memory and Language.

